# Functional Remodeling of the Contractile Smooth Muscle Cell Cortex, a Provocative Concept, Supported by Direct Visualization of Cortical Remodeling

**DOI:** 10.3390/biology11050662

**Published:** 2022-04-26

**Authors:** Worawit Suphamungmee, William Lehman, Kathleen G. Morgan

**Affiliations:** 1 Department of Anatomy, Faculty of Science, Mahidol University, Rama VI Road, Bangkok 10400, Thailand; worawit.sup@mahidol.ac.th; 2 Department of Health Sciences, Boston University Sargent College, 635 Commonwealth Ave., Boston, MA 02215, USA; 3 Department of Physiology & Biophysics, Boston University School of Medicine, 700 Albany Street, Boston, MA 02118, USA

**Keywords:** actin, zyxin, talin, immunoelectron microscopy, vascular smooth muscle

## Abstract

**Simple Summary:**

As a key element of the smooth muscle cell contractile apparatus, the actin cytoskeleton participates in the development of force by acting as a molecular track for the myosin cross bridge motor. At the same time, the actin cytoskeleton must transmit the force developed during contraction to the extracellular matrix and, thus, to neighboring cells. This propagation of force to the cell periphery and beyond is initiated in part on specifically localized cellular cortical actin filaments also involved in mechano-chemical transduction. During the contractile process itself and in response to extracellular structural and chemical alterations, the smooth muscle actin cytoskeletal remodels. This indicates that the cytoskeleton is a dynamic cellular organelle that adapts to the changes in cell shape and chemical cues. Current evidence connecting contractile function and mechano-transduction mechanisms to the plasticity of the vascular smooth muscle actin cytoskeleton is reviewed; we then describe new evidence for cytoskeletal remodeling in vascular smooth muscle cells. Here, using immunoelectron microscopy, we visualize the actin binding proteins filamin A, zyxin and talin in these cells and show that they participate in the cortical cell cytoskeletal alteration, thus supporting the premise that smooth muscle cell remodeling occurs during contraction.

**Abstract:**

Considerable controversy has surrounded the functional anatomy of the cytoskeleton of the contractile vascular smooth muscle cell. Recent studies have suggested a dynamic nature of the cortical cytoskeleton of these cells, but direct proof has been lacking. Here, we review past studies in this area suggesting a plasticity of smooth muscle cells. We also present images testing these suggestions by using the technique of immunoelectron microscopy of metal replicas to directly visualize the cortical actin cytoskeleton of the contractile smooth muscle cell along with interactions by representative cytoskeletal binding proteins. We find the cortical cytoskeletal matrix to be a branched, interconnected network of linear actin bundles. Here, the focal adhesion proteins talin and zyxin were localized with nanometer accuracy. Talin is reported in past studies to span the integrin–cytoplasm distance in fibroblasts and zyxin is known to be an adaptor protein between alpha-actinin and VASP. In response to activation of signal transduction with the alpha-agonist phenylephrine, we found that no movement of talin was detectable but that the zyxin-zyxin spacing was statistically significantly decreased in the smooth muscle cells examined. Contractile smooth muscle is often assumed to have a fixed cytoskeletal structure. Thus, the results included here are important in that they directly support the concept at the electron microscopic level that the focal adhesion of the contractile smooth muscle cell has a dynamic nature and that the protein–protein interfaces showing plasticity are protein-specific.

## 1. Background

The cortical actin cytoskeleton is an essential participant in a variety of physiological processes in virtually all cells. As pointed out in this and other articles of this Special Issue covering “Reviews in Actin Cytoskeleton Dynamics”, the complex and varied cortical cellular filament apparatus is vital for communicating intracellular chemical and mechanical signals to the extracellular matrix and, conversely, extracellular perturbations to a cell’s cytoplasm (chemo- and mechano-transduction). The cytoskeleton not only responds to environmental cues to maintain cellular homeostasis, but it also plays a structural role in many cells as well as participating in cellular motility. In turn, cytoskeletal defects, and corresponding imbalances in chemo- and mechano-transduction, are associated with maladaptive remodeling and the onset of the development of well-known diseases, such as familial thoracic aortic aneurysms and dissection caused by mutations of smooth muscle alpha-actin, leading to other vascular disease linkages, (e.g., coronary artery disease and stroke) [1]. Dysfunction of mechano-sensing proteins may also result in partial or total disruption in mechano-transductive pathways of vascular smooth muscle, together with decreased contractile function and loss of the regulation of blood pressure [2].

It is well accepted that the smooth muscle cortical cytoskeleton such as that in all somatic cells responds to multiple chemical and mechanical cues that also affect smooth muscle cellular growth and differentiation status as well as their responsiveness to agonists and antagonists. However, unlike actin filaments in skeletal muscle cells that are almost entirely devoted to contractility, the actin filaments in smooth muscle are likely to have widely different roles distinct from those that only affect contractility. Importantly, the dynamic contributions of the cytoskeleton of smooth muscle cells (the subject of this review) are particularly difficult to unravel since the architectural and functional dividing line between cytoskeletal and contractile actin is uncertain. Moreover, many studies on the smooth muscle cytoskeleton have used and characterized smooth muscle cells in culture as their model. It is important to remember that these cultured cells may be at various stages of dedifferentiation further complicate the interpretation of the results. In contrast, our group has studied, in addition to contractile tissue, freshly isolated, differentiated, and fully contractile smooth muscle cells.

Finally, regardless of their source, cytoskeletal and contractile actin filaments typically do not act alone [3], and their function is defined by a host of actin binding proteins which act to specifically localize their cellular site of action and modulate their behavior and activity [4,5,6]. Again, discriminating between the action of actin binding proteins in smooth muscle cells is challenging because of the dual nature of smooth muscle cell function, at once associated with non-contractile cell function while also displaying contractile attributes to function as a muscle. Seminal structural and biochemical studies we describe below aim to differentiate between contractile and cytoskeletal domains of smooth muscle cells, which provides a foundation for understanding the dynamics of actin binding protein redistribution following agonist stimulation.

## 2. Organization of Actin Filaments in the Vascular Smooth Muscle Cell

In striated muscle, the force generated by the cross bridges of the contractile filaments in the sarcomeres is transmitted to tendons [7]. In contrast, smooth muscle lacks clear sarcomeres and how the forces generated by cross bridges in the interior of the cell are transmitted to the transmembrane integrins that communicate with the extracellular matrix and the outside world is a continuing area of investigation. Here, we review past studies in the area of the composition and function of the cortical cytoskeleton of the mammalian vascular smooth muscle cell and the implications of these studies for the plasticity of the smooth muscle cell. We also include novel, unpublished, images we have obtained with the use of immunoelectron microscopy of metal replicas to directly visualize the cytoskeletal binding proteins and their interactions with actin in the cortex of the cell.

In 1994, North et al. [8] presented a novel model for the cytoskeleton of chicken gizzard smooth muscle cells where the contractile filaments were shown to run diagonally in the contractile smooth muscle cells from the dense bodies in the core of the cell to the adhesion plaques (also called dense plaques, or focal adhesions) at the cell surface. In the North et al. work, alpha actin was described as being part of the main contractile unit, while the dense bodies were linked by beta non-muscle actin linearly down the length of the cell. Gamma actin was not imaged due to lack of a specific antibody at that time. Furthermore, at that time, since only static structures were examined, the functional dynamics of the smooth muscle cytoskeleton apparatus could not be related to mechano-transduction principles.

More recently, immunofluorescence studies at the subcellular level in mammalian vascular smooth muscle cells (VSMC) suggest some significant differences from this model. All three actin isoforms present in contractile vascular smooth muscle cells have now been directly immunolocalized with deconvolution microscopy at the light microscope level. Alpha smooth muscle actin is now known to be confined to the contractile filament bundles in the core of the cell and beta actin is associated with dense bodies, consistent with the North model, but the beta actin isoform is also found at the focal adhesions at the cell edge, referred to here as the cell cortex. Gamma non-muscle actin is primarily located in the cortex of the cell, near the cell membrane [9]. Alpha actin as well as beta actin are relatively stable and do not show detectable changes in polymerization with alpha-adrenergic stimulation by an agonist such as phenylephrine (PE). In contrast, PE triggers an increased actin polymerization of gamma non-muscle actin as well as an endosome-dependent recycling of the focal adhesion protein zyxin [9,10,11]. The agonist stimulation of VSMCs activates a signaling cascade which may increase actomyosin cross-bridge cycling and generate mechanical force. As a consequence, the force increases the tension on non-muscle actin in which some isoforms are associated with focal adhesion proteins, such as focal adhesion kinase (FAK), talin, vinculin and paxillin and to integrins. However, the functional significance of the agonist-induced changes in the structure of the non-muscle actin cytoskeleton in the living smooth muscle cell has not been clear.

Smooth muscle cells contain four isoforms of the actin: alpha smooth muscle actin, beta non-muscle actin, gamma smooth muscle actin and gamma non-muscle actin [12,13]. These isoforms are products of the separate genes with different expression patterns and different functions. Surprisingly, they share quite similar protein sequences, almost 93% identical to each other [13,14]. While alpha smooth muscle and gamma smooth muscle actin are the predominant isoforms in the contractile filaments of VSMC, the potential roles of beta non-muscle and gamma non-muscle actin are less clearly defined. Previous deconvolution immunofluorescence microscopy of the vascular smooth muscle structure demonstrated the presence of gamma non-muscle actin specifically restricted to the cell cortex [15] though the precise function of the cortical non-muscle actin remains to be defined in detail. Alpha smooth muscle actin, however, clearly appears in the contractile domain of the cytoplasm. Beta actin is found in prominent association with dense bodies as well as focal adhesion proteins. It is suggested that beta actin functions to attach contractile filaments at dense bodies and transmit contractile forces generated in the core of the cell to the focal adhesions at the surface of the cell and then to the inter-cellular space. Non-muscle gamma actin is concentrated in filaments at the cell cortex but its functions are still yet to be clearly defined.

**Figure 1:**
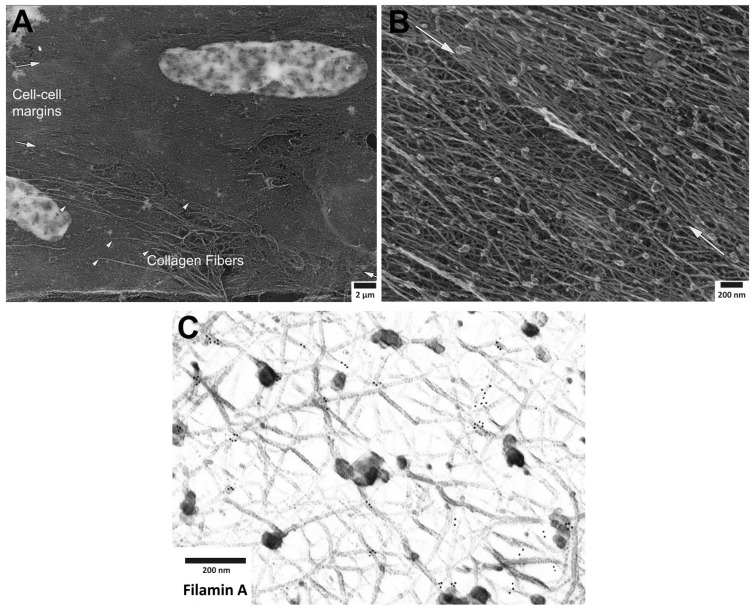
Cortical actin cytoskeleton of vascular smooth muscle at increasing levels of magnification. (**A**) Low magnification view of two adherent vascular smooth muscle cells, defined by central nuclei in each cell and collagen fibers (arrowheads), especially prominent at cell-to-cell margins. (**B**) Higher magnification of cortical actin cytoskeleton; white arrows denoting cell-to-cell margins of two adherent enzymatically isolated cells. (**C**) Further increase in magnification reveals details of a branched, interconnecting actin cytoskeleton. Gold beads mark immunoEM labeling of filamin A at the branch points of actin filaments. See text for details and Appendix A for materials and methods.

Recently, we have investigated the structural disposition and the possibility of remodeling of VSMC actin filaments and select actin binding proteins by immunoelectron microscopy (EM) of the smooth muscle cytoskeleton, specifically focusing on the cortex of the cell with the method of metal replica imaging. We did this as a means to test and refine the concept of a dynamic cortical cytoskeleton in the smooth muscle cell. The complexity of the inter-weaving fabric of the smooth muscle cytoskeleton can be appreciated by examination of Figure 1, where the Triton-extracted actin cytoskeleton is shown at sequentially increasing levels of magnification. Despite the complexity of this structure, it is clear that an alpha-adrenergic agonist, such as phenylephrine (PE), produces defined remodeling of the cytoskeletal connections to the alpha actinin-VASP adaptor protein, zyxin. In contrast, no remodeling of the cytoskeletal connections to the focal adhesion protein talin occurs. Thus, in this study, agonist-induced remodeling of the contractile smooth muscle cell cytoskeleton is confirmed at the EM level and is shown to be selective and protein-specific. Since contractile smooth muscle cells, unlike migrating, proliferating smooth muscle cells are often assumed to have a fixed cytoskeletal structure, these results are important in that they indicate directly, a dynamic nature of the cortical cytoskeleton of the differentiated, contractile smooth muscle cell.

## 3. Roles of Adhesion Proteins on the Cortical Actin

**Figure 2:**
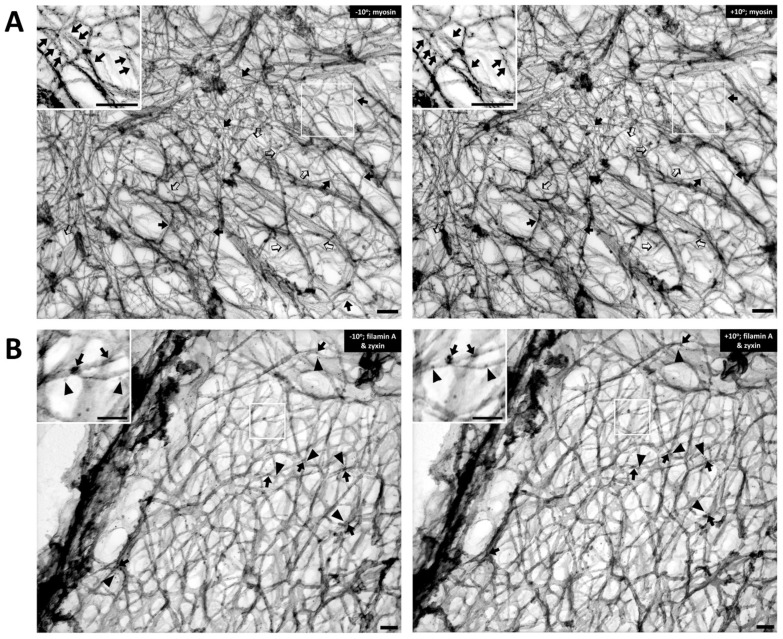
The cortical actin cytoskeleton of contractile VSMC consists of a branched network of groups of linear actin bundles containing filamin A and zyxin. Tilt-paired images of S1-decorated cortical actin (**left**, −10° tilted image; **right**, +10° tilted image). (**A**) Labeling of filaments with myosin S1. Arrows = myosin S1. Bar = 100 nm. Depth estimation is 160–180 nm. Actin filaments are Triton X-100 extracted, labeled with myosin S1 and metal cast. Inset shows S2 myosin “arrowheads” (marked here with arrows). Both orthogonal 90-degree (solid arrows) and 60-degree (open arrows) branches are seen in the rest of the figure, suggesting filamin A-mediated cross-linking and Arp2/3-mediated branching, respectively. (**B**) Double labeling of filamin A and zyxin (**left**, −10° tilted image; **right**, +10° tilted image). Arrows = 10 nm beads of anti-filamin A labeling. Arrowheads = 6-nm beads of anti-zyxin labeling. Bar = 100 nm. Depth estimation is 180–200 nm. Inset shows higher magnification of actin cytoskeleton where filamin A (arrows) binds mostly at the crosslinks but zyxin (arrowheads) is located about 20–30 nm away from the crosslink. Some of the black arrows are on a filament that is out of the major plane of the image.

Cortical actin supports the vascular smooth muscle cell morphology by bridging beta integrin at the cell membrane and focal adhesion proteins to the contractile elements inside the cell [16,17,18,19]. This cell type is highly dynamic and undergoes remodeling by the complex interactions of actin binding proteins and focal adhesion proteins. As is shown in Figure 2A, the Triton-extracted, cortical, actin cytoskeleton of the contractile VSMC is a branched network of groups of linear actin bundles. Apparent 60–65 degree (open arrows) and 90 degree (solid arrows) branches are seen, which suggest linkages to Arp2/3-capped daughter filaments or filamin A cross-connections, respectively [20,21]. The presence of orthogonal cross-linking in the cortex suggests an active role of filamin A, possibly in determining the structure and function of the cortical actin cytoskeleton [22]. Although we did not observe the classic 70-degree branches associated with Arp2/3 [20,23,24], it seems likely that if there are mechanical pushing or pulling forces generated within the cortical cellular cytoskeleton, it is possible that Arp2/3 mechanisms could be associated with angles other than the classic 70 degrees. Further investigations are needed to address questions concerning the dynamic change of the newly branched actin cytoskeleton at the cell cortex when there is intracellular, mechanical force induced by agonist stimulation. With respect to filamin A-induced cross-linking, it is also important to rule out the possibility that these filaments are simply lying on top of each other.

To test the idea that these orthogonal structures are due to filamin A-induced cross-linking of actin filaments, rather than simply overlying unconnected filaments, we immunostained metal replicas of the cortical cytoskeleton from contractile VSMCs for filamin A (arrows, Figure 2B). For comparison, we co-stained the same sections with anti-zyxin antibodies (arrowheads, Figure 2B). Zyxin is an adaptor protein that has been reported to link VASP with alpha-actinin at focal adhesions [23,25]. VASP is reported to drive linear actin polymerization in tetrameric bundles and has been shown to be involved in actin polymerization in VSMC [26,27]. In the inset of Figure 2B, filamin A (solid arrows, 10 nm beads) is seen at points of orthogonal branches, as predicted. The fact that zyxin is known to bind proteins other than actin (VASP and alpha-actinin) and, thus, is not expected to directly bind actin, is consistent with the apparent lack of direct association of the zyxin beads (arrowheads) with actin filaments.

Zyxin has the postulated function to serve as a structural protein as well as a modulator to stretch-induced gene expression in VSMC. When exposed to cyclic stretch, zyxin rapidly translocates from the focal adhesion multiprotein complex to the nucleus and alters the expression of mechano-sensitive genes [28,29]. This mechanism implies a role for zyxin in transducing mechanical stimuli from the cell membrane to the nucleus of VSMC. Thus, current evidence is consistent to its responsive role to mechanical cues [28,30]. Direct observation of zyxin-actin interaction has not been reported. A study of zyxin co-culture with a barbed end-capping protein revealed its recruitment to the free barbed ends suggesting the linkage of zyxin to actin polymerizing and associated proteins, such as VASP. Although zyxin is one of the focal adhesion proteins involved in the remodeling of the actin cytoskeleton, the interaction between zyxin and other proteins and how zyxin contributes to remodeling mechanisms remains unclear.

During contractile activity, the presence of talin in focal adhesions also leads to the recruitment of vinculin, further stabilizing the focal adhesion complex [31]. Fluorescence microscopic analysis has demonstrated the fluctuating length of talin in a range of 100–350 nm in living CV1 cells [32]. In vivo measurement of talin extension with over-expression of the vinculin head revealed a tight binding of the vinculin head that promotes talin stretching. However, the expected amplitude of change is suppressed implying a subsequent relaxation of the force in the talin-mediated force transmission pathway. Stability of vinculin at focal adhesions primarily depends on the binding with talin, whereas the binding to F-actin filaments is also affected by the impact due to the force load [33]. Both a decrease of actomyosin-based force load to focal adhesions or an imposed deficiency in vinculin expression revealed delocalization of vinculin from adhesion sites [34,35].

**Figure 3:**
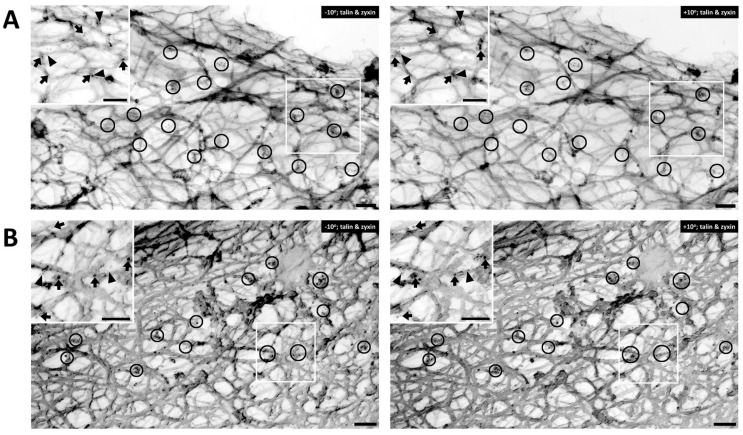
Talin versus zyxin labeling in response to agonist activation. (**A**) Double labeling of talin and zyxin (**left**, −10° tilted image; **right**, +10° tilted image) in unstimulated VSMC. Arrows = 10 nm beads, anti-talin labeling. Arrowheads = 6 nm beads of anti-zyxin labeling. Bars = 100 nm. Depth estimation is 160–180 nm. (**B**) Double labeling of talin and zyxin (**left**, −10° tilted image; **right**, +10° tilted image) in phenylephrine-stimulated VSMC. Arrows = 10 nm beads of anti-talin labeling. Arrowheads = 6 nm beads of anti-zyxin labeling. Bars = 100 nm. Depth estimation is 200–220 nm. Clusters of talin and zyxin are shown in the black circles. In both panels, the inset shows a larger view of actin cytoskeleton in the VSMC where talin (arrows) and zyxin (arrowheads) colocalized close to actin filaments.

**Figure 4:**
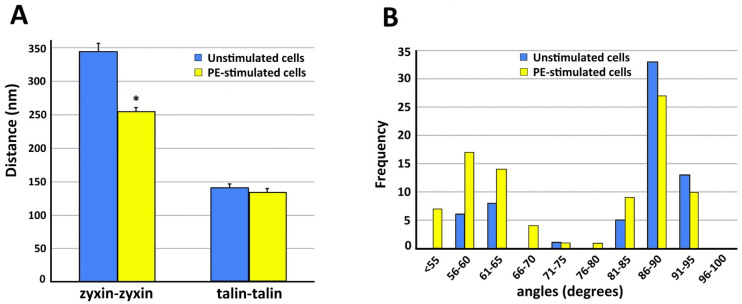
Quantitation of structural features of VSMC cortical actin cytoskeleton. In all panels, analysis is from five different cells in each category. Data = mean ± standard error with statistical significance, *p* < 0.05 (*). (**A**) Spacing between zyxin beads is significantly decreased with PE stimulation. Talin spacing is not significantly different. (**B**) Frequency distribution of angles measured from actin branches in VSMC.

In contractile VSMCs, talin is known to be an abundant cytoskeleton protein associated with focal adhesions and has recently been described to be a “molecular ruler” to define the size of focal adhesions [36]. The cells shown in Figure 3 are co-immunostained with talin and zyxin. Similar to filamin A, talin is found mostly in the cortical area of the cell. Figure 3A displays an unstimulated cell, whereas Figure 3B shows a cell activated with PE. In both figure panels talin (arrows) and zyxin (arrowheads) are colocalized close to actin filaments. Clusters of talin and zyxin are indicated by circles. The distance between beads was quantified except in areas where superimposed filaments and image depth prevented accurate measurement. In the unstimulated cell shown in Figure 3A the distance between beads for talin is 120–200 nm and in Figure 3B, with PE activation, the distance is a very similar (100–200 nm in range), indicating that talin and zyxin are closely interacted. The distance between talin beads is rather in a fixed position and not perturbed by the activation of a vasoconstrictor agonist such PE. In contrast, the distance between zyxin beads in the unstimulated cell shown in Figure 3A is 300–400 nm but that in the presence of PE is 200–300 nm. These differing results with different proteins suggest that the apparent decreased zyxin–zyxin distance in the presence of PE is likely due to a cytoskeletal remodeling event rather than simply the shortening of the cell. This is confirmed by the statistical analysis shown in Figure 4. As is shown in Figure 4A, in the presence of PE the zyxin–zyxin spacing decreases in a statistically significant manner but the talin–talin spacing is not significantly changed. Functionally, this suggests that the lack of change in talin spacing would maintain a constant size of the focal adhesion, as has been reported for contractile VSMCs [37].

## 4. Dynamics of Smooth Muscle Cell Cytoskeleton

These findings are also of interest in the context of the recently proposed Kanchanawong model for focal adhesion architecture [36,38], based on super-resolution microscopy of fibroblasts, in which talin is seen to span the focal adhesion, stretching vertically between the extracellular matrix and the intracellular cytoplasm, with no evidence of moving laterally within the focal adhesion. In contrast, in the Kanchanawong et al. model, zyxin has been shown to be restricted to the inner edge of the focal adhesion, farthest from the cell membrane, and to connect VASP to alpha actinin. In contractile VSMCs [11] in the presence of the vasoconstrictor, PE, zyxin moves between a membrane fraction and a cytoskeletal fraction, cycling in and out of endosomes. We suggest that this allows remodeling of the zyxin/actin attachments to the FA in VSMCs. In the present study, we have confirmed the mobility of zyxin within the submembranous cortical cytoskeleton at the EM level.

Additional observations that emerge from a quantitative analysis of these images indicate that the frequency distribution of the angles associated with the apparent actin branching or cross-linking changes in the presence of PE (Figure 4B). When the frequency distributions of the actin angles are compared in the presence and absence of PE, it is notable that in unstimulated cells orthogonal actin branches (90°) are abundant but 60–65° angles are rare. After PE-stimulation, orthogonal branches are also common but 60–65° branches are increased in frequency, likely reflecting the increased remodeling of the non-muscle actin cytoskeleton by an Arp2/3-based mechanism.

**Figure 5:**
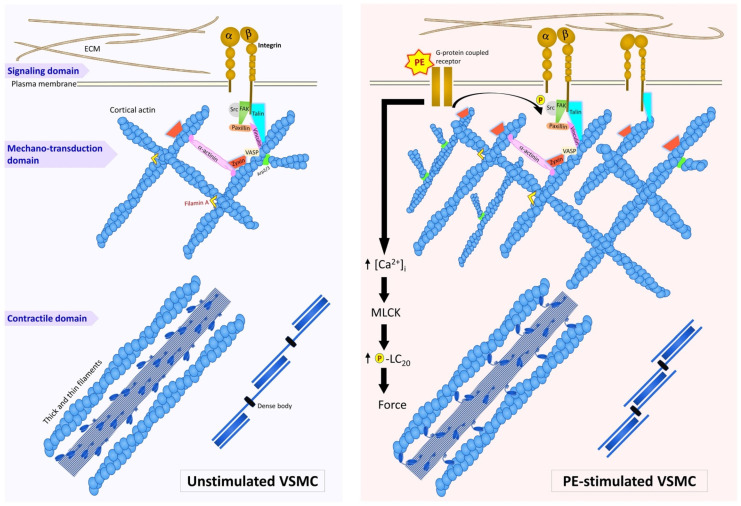
Protein-specific cytoskeletal remodeling of VSMC. **Left**: Cell at resting condition. **Right**: Cell in response to a contractile agonist, phenylephrine (PE). Please note: We have examined the outermost cortical surface of single smooth muscle cells isolated from vascular tissue that are then absorbed onto coverslips. Unlike cultured smooth muscle cells with morphologically distinct ventral and dorsal surfaces, the cells examined here are representative of cells embedded in the vascular tissue wall, which likely are isotropically surrounded by the extracellular matrix (ECM). Thus, the schematic does not designate a ventral or dorsal cell surface.

The proliferative, migratory VSMC is known to be able to remodel its cytoskeleton, creating new focal adhesions at the leading edge and dissolving focal adhesions at the trailing edge (reviewed in [39,40]). In contrast, until recently, it was thought that the contractile VSMC cytoskeleton is a static structure, much as the skeletal or cardiac muscle cell myofibrils have been traditionally viewed. However, recent studies, reviewed here, have shown that contractile VSMCs have a dynamic nature and are capable of undergoing agonist-induced cytoskeletal rearrangements [41]. Importantly, the rearrangements of protein–protein interfaces are protein-specific. These cytoskeletal remodeling events, in turn, can lead to changes in contractile force and stiffness [42]. We additionally show here, for the first time, at the EM level, supporting evidence for the view that the cortical cytoskeleton of the contractile VSMC displays selective plasticity of cytoskeletal elements. It has been frequently demonstrated that exposure to vasoconstrictors such as PE increases intracellular Ca^2+^ and myosin phosphorylation levels to increase contractile force, and we have now summarized these concepts in the diagram in Figure 5, adding on the additional novel findings that PE also causes a cytoskeletal rearrangement of zyxin associated with the juncture of the actin cytoskeleton and the focal adhesions in vascular smooth muscle cells. Under the same conditions, it is also known that actin polymerization is activated by VASP and that cellular stiffness increases [11,43].

Interestingly, this plasticity is protein-specific since, at the same time talin, displays no detectable remodeling at the EM level. These results confirm the generally held, but until now only superficially demonstrated, concept that vascular smooth muscle functions at an intermediate point between structurally dynamic non-muscle cells and structurally fixed striated muscle cells. Taking all recent findings together, we anticipate our preparations of isolated smooth muscle cells will prove to be ideal sample to examine the dynamics of their cortical actin domain architecture at atomic resolution by cryo-electron tomography, particularly given recent advances in focused ion beam methodology [44,45,46,47].

## 5. Conclusions and Perspective

Here, we have reviewed previous studies looking at the smooth muscle cortical actin cytoskeleton and have concluded that it is a dynamic structure. Furthermore, we have additionally provided new evidence supporting this characterization. The dynamics we describe here allow bundles of actin filaments as well as individual filaments to grow in length and to shorten, and, thus, to accommodate to cell length changes as well as to allow changes in the stiffness of the cells and the smooth muscle tissues where the cells reside. The corresponding remodeling of the cortical cytoskeleton is in large part a response to filament interactions with numerous actin-binding proteins. In turn, changes in filament structure and their corresponding binding-protein associations affect signaling pathways and, consequently, communicate intracellular events to focal adhesions and the extracellular matrix possibly in a load-dependent manner (Figure 5). In this review, we provide evidence for the mobility of the focal adhesion protein zyxin and lack of mobility of talin at the cortical actin domain of the smooth muscle cell during cell excitation, providing direct evidence of the plasticity of the smooth muscle cell cortex.

To date, most structural studies on the smooth muscle cortical cytoskeleton, including our own, were carried out at the light microscope level or by means of traditional transmission electron microscopy, in each case, coupled with immunolocalization of key proteins. In the future, we anticipate preparations of isolated smooth muscle cells will be ideal to examine the dynamics of the cortical actin domain architecture at near atomic resolution by cryo-electron tomography, particularly given recent progress in focused ion beam methodology. We, thus, anticipate further advances in understanding dynamics of the smooth muscle cortical assembly.

## Data Availability

Not applicable.

## Appendix A. Materials and Methods

### Appendix A.1. Preparation of Smooth Muscle Cells

All procedures in this study were performed according to protocols approved by the Institutional Care and Use Committee and comply with the NIH Guide for the Care and Use of Laboratory Animals, approved number D16-00204 (A3316-01). Portal veins were taken from young ferrets (3 months old, male, Marshall Farms, North Rose, NY, USA). Ferret tissues were used because they are generally assumed to be the best non-primate model for the human vascular system [48]. After the animals were euthanized by an overdose of isoflurane, the portal vein was quickly removed and immediately placed in an oxygenated physiological saline (PSS) buffer, as previously described [9]. Dissection was performed in PSS to remove fat, adventitia and endothelial cells. Portal vein tissue (16 mg) was cut into 2 × 2 mm^2^ pieces and transferred to an enzyme cocktail containing 259 U/mL CLS 2 Collagenase (Worthington Biochemical, Lakewood, NJ, USA), 3.38 U/mL Elastase (Grade II, 3.65 U/mg; Boehringer Mannheim, Mannheim, Germany) and 0.26 mg/mL Soybean Trypsin inhibitor (type II-S, Sigma, Kawasaki, Japan) in Ca^2+^/Mg^2+^-free Hank’s balanced salt solution (HBSS) with 0.075% BSA added [2]. To dissociate cells from the extracellular matrix, tissues were agitated in an enzyme cocktail for 40 min at 34 °C with oxygenation (95% O_2_ and 5% CO_2_). Tissue was rinsed with 10 mL HBSS/BSA and sequentially transferred to second and third set of the same enzyme ingredients but containing half amount of collagenase for 20 min of incubation time for each set. After the last enzyme incubation, tissues were rinsed with HBSS/BSA and were taken for evaluation under an optical microscope. The cell solution was gently poured onto cleaned 22 × 22 mm^2^ coverslips in 6-well plates which were placed on ice with O_2_ bubbling to allow them to settle while gradually adding back Ca^2+^ and Mg^2+^ to reach the final concentrations of 1 mM and 1.2 mM, respectively. 0.1 mM PE was added to some cells for 10 min to activate contractility of smooth muscle cells. For unstimulated cells, no PE was added.

### Appendix A.2. Immunoelectron Microscopy and Application of Metal Replica Methodology

Immunoelectron microscopy was carried out by a modification of previous methods [49]. Cytoskeleton preparations were made from freshly dissociated smooth muscle cells. In brief, the cell cytoskeleton was extracted in 0.75% Triton X-100 in PHEM buffer (60 mM PIPES, 25 mM HEPES, pH 6.9, 10 mM EGTA, 2 mM MgCl_2_), for 2 min, 37 °C. After washing with PHEM, the cytoskeleton was fixed in 1% glutaraldehyde in PHEM for 10 min.

The cytoskeletons were treated with 1 mg/mL sodium borohydride, for 10 min to remove excessively reactive aldehydes followed by blocking with 0.5% BSA /PBS for 2 h at RT. Primary antibodies were diluted in 0.5% BSA/PBS and left incubated for overnight at 4 °C. The cytoskeletons were left in secondary antibodies for 4 h at room temperature followed by several washes in PBS and then fixed in 1% glutaraldehyde.

After immunolabeling, the cytoskeletons were rapidly frozen on a helium-cooled copper block, freeze-dried at −85 °C in a freezing apparatus (Cressington CFE-50; Cressington, Watford, UK), and rotary coated with 1.4 nm of tungsten-tantalum followed by 3 nm of carbon without rotation. Replicas were floated off coverslips using 25% hydrofluoric acid, washed in water and collected on 200-mesh copper grids coated with formvar. Tilted pair negatives (+/−10°) were collected in a JEOL 1200-EX transmission electron microscope at 100 kV and stereo-pairs recorded in order to visualize the actin cytoskeleton of smooth muscle cells in 3-D.

### Appendix A.3. Antibodies

ImmunoEM: Either goat polyclonal anti-zyxin (1:100)/mouse monoclonal anti-filamin A (1:100) or goat polyclonal anti-zyxin (1:100)/mouse monoclonal anti-talin (1:100) were used as primary antibodies for double labeling. Secondary antibodies were 6 nm colloidal gold-conjugated donkey anti-goat IgG and 10 nm colloidal gold-conjugated donkey anti-mouse IgG.

## References

[1] Guo D.C., Papke C.L., Tran-Fadulu V., Regalado E.S., Avidan N., Johnson R.J., Kim D.H., Pannu H., Wjlling M.C., Sparks E. (2009). Mutations in smooth muscle alpha-actin (ACTA2) cause coronary artery disease, stroke, and Moyamoya disease, along with thoracic aortic disease. Am. J. Hum. Genet..

[2] Milewicz D.M., Guo D.C., Tran-Fadulu V., Lafont A.L., Papke C.L., Inamoto S., Kwartler C.S., Pannu H. (2008). Genetic basis of thoracic aortic aneurysms and dissections: Focus on smooth muscle cell contractile dysfunction. Annu. Rev. Genom. Hum. Genet..

[3] Gunning P.W., Ghoshdastider U., Whitaker S., Popp D., Robinson R.C. (2015). The evolution of compositionally and functionally distinct actin filaments. J. Cell Sci..

[4] Winder S.J., Ayscough K.R. (2005). Actin-binding proteins. J. Cell Sci..

[5] Pollard T.D. (2016). Actin and actin-binding proteins. Cold Spring Harb. Perspect. Biol..

[6] Kadzik R.S., Homa K.E., Kovar D.R. (2020). F-Actin cytoskeleton network self-organization through competition and cooperation. Annu. Rev. Cell Dev. Biol..

[7] Rassier D.E., MacIntosh B.R., Herzog W. (1999). Length dependence of active force production in skeletal muscle. J. Appl. Physiol..

[8] North A.J., Gimona M., Lando Z., Small J.V. (1994). Actin isoform compartments in chicken gizzard smooth muscle cells. J. Cell Sci..

[9] Kim H.R., Gallant C., Leavis P.C., Gunst S.J., Morgan K.G. (2008). Cytoskeletal remodeling in differentiated vascular smooth muscle is actin isoform dependent and stimulus dependent. Am. J. Physiol. Cell Physiol..

[10] Exton J.H. (1985). Mechanisms involved in alpha-adrenergic phenomena. Am. J. Physiol..

[11] Poythress R.H., Gallant C., Vetterkind S., Morgan K.G. (2013). Vasoconstrictor-induced endocytic recycling regulates focal adhesion protein localization and function in vascular smooth muscle. Am. J. Physiol. Cell Physiol..

[12] Drew J.S., Moos C., Murphy R.A. (1991). Localization of isoactins in isolated smooth muscle thin filaments by double gold immunolabeling. Am. J. Physiol..

[13] Perrin B.J., Ervasti J.M. (2010). The actin gene family: Function follows isoform. Cytoskeleton.

[14] Vedula P., Kurosaka S., Leu N.A., Wolf Y.I., Shabalina S.A., Wang J., Sterling S., Dong D.W., Kashina A. (2017). Diverse functions of homologous actin isoforms are defined by their nucleotide, rather than their amino acid sequence. eLife.

[15] Gallant C., Appel S., Graceffa P., Leavis P., Lin J.J., Gunning P.W., Schevzov G., Chaponnier C., DeGnore J., Lehman W. (2011). Tropomyosin variants describe distinct functional subcellular domains in differentiated vascular smooth muscle cells. Am. J. Physiol. Cell Physiol..

[16] Ingber D.E. (2002). Mechanical signaling and the cellular response to extracellular matrix in angiogenesis and cardiovascular physiology. Circ. Res..

[17] Gunst S.J., Zhang W. (2008). Actin cytoskeletal dynamics in smooth muscle: A new paradigm for the regulation of smooth muscle contraction. Am. J. Physiol. Cell Physiol..

[18] Zaidel-Bar R. (2009). Evolution of complexity in the integrin adhesome. J. Cell Biol..

[19] Rossier O., Octeau V., Sibarita J.B., Leduc C., Tessier B., Nair D., Gatterdam V., Destaing O., Albigès-Rizo C., Tampé R. (2012). Integrins β1 and β3 exhibit distinct dynamic nanoscale organizations inside focal adhesions. Nat. Cell Biol..

[20] Mullins R.D., Heuser J.A., Pollard T.D. (1998). The interaction of Arp2/3 complex with actin: Nucleation, high affinity pointed end capping, and formation of branching networks of filaments. Proc. Natl. Acad. Sci. USA.

[21] Nakamura F., Stossel T.P., Hartwig J.H. (2011). The filamins: Organizers of cell structure and function. Cell Adhes. Migr..

[22] Lamsoul I., Dupré L., Lutz P.G. (2020). Molecular tuning of filamin A activities in the context of adhesion and migration. Front. Cell Dev. Biol..

[23] Crawford A.W., Michelsen J.W., Beckerle M.C. (1992). An interaction between zyxin and alpha-actinin. J. Cell Biol..

[24] Svitkina T.M., Borisy G.G. (1999). Arp2/3 complex and actin depolymerizing factor/cofilin in dendritic organization and treadmilling of actin filament array in lamellipodia. J. Cell Biol..

[25] Drees B., Friederich E., Fradelizi J., Louvard D., Beckerle M.C., Golsteyn R.M. (2000). Characterization of the interaction between zyxin and members of the Ena/vasodilator-stimulated phosphoprotein family of proteins. J. Biol. Chem..

[26] Kim H.R., Graceffa P., Ferron F., Gallant C., Boczkowska M., Dominguez R., Morgan K.G. (2010). Actin polymerization in differentiated vascular smooth muscle cells requires vasodilator- stimulated phosphoprotein. Am. J. Physiol. Cell Physiol..

[27] Wang Y.X., Wang D.Y., Guo Y.C., Guo J. (2019). Zyxin: A mechanotransductor to regulate gene expression. Eur. Rev. Med. Pharmacol. Sci..

[28] Cattaruzza M., Lattrich C., Hecker M. (2004). Focal adhesion protein zyxin is a mechanosensitive modulator of gene expression in vascular smooth muscle cells. Hypertension.

[29] Ghosh S., Kollar B., Nahar T., Babu S.S., Wojtowicz A., Sticht C., Gretz N., Wagner A.H., Korff T., Hecker M. (2015). Loss of the mechanotransducer zyxin promotes a synthetic phenotype of vascular smooth muscle cells. J. Am. Heart Assoc..

[30] Yoshigi M., Hoffman L.M., Jensen C.C., Yost H.J., Beckerle M.C. (2005). Mechanical force mobilizes zyxin from focal adhesions to actin filaments and regulates cytoskeletal reinforcement. J. Cell Biol..

[31] Klapholz B., Brown N.H. (2017). Talin—The master of integrin adhesions. J. Cell Sci..

[32] Margadant F., Chew L.L., Hu X., Yu H., Bate N., Zhang X., Sheetz M. (2011). Mechanotransduction in vivo by repeated talin stretch-relaxation events depends upon vinculin. PLoS Biol..

[33] Peng X., Nelson E.S., Maiers J.L., DeMali K.A. (2011). New insights into vinculin function and regulation. Int. Rev. Cell Mol. Biol..

[34] Zhang X., Jiang G., Cai Y., Monkley S.J., Critchley D.R., Sheetz M.P. (2008). Talin depletion reveals independence of initial cell spreading from integrin activation and traction. Nat. Cell Biol..

[35] Pasapera A.M., Schneider I.C., Rericha E., Schlaepfer D.D., Waterman C.M. (2010). Myosin II activity regulates vinculin recruitment to focal adhesions through FAK-mediated paxillin phosphorylation. J. Cell Biol..

[36] Liu J., Wang Y., Goh W.I., Goh H., Baird M.A., Ruehland S., Teo S., Bate N., Critchley D.R., Davidson M.W. (2015). Talin determines the nanoscale architecture of focal adhesions. Proc. Natl. Acad. Sci. USA.

[37] Saphirstein R.J., Gao Y.Z., Lin Q.Q., Morgan K.G. (2015). Cortical actin regulation modulates vascular contractility and compliance in veins. J. Physiol..

[38] Kanchanawong P., Shtengel G., Pasapera A.M., Ramko E.B., Davidson M.W., Hess H.F., Waterman C.M. (2010). Nanoscale architecture of integrin-based cell adhesions. Nature.

[39] Burridge K., Fath K., Kelly T., Nuckolls G., Turner C. (1988). Focal adhesions: Transmembrane junctions between the extracellular matrix and the cytoskeleton. Annu. Rev. Cell Biol..

[40] Zhang W., Gunst S.J. (2008). Interactions of airway smooth muscle cells with their tissue matrix: Implications for contraction. Proc. Am. Thorac. Soc..

[41] Ohanian J., Pieri M., Ohanian V. (2015). Non-receptor tyrosine kinases and the actin cytoskeleton in contractile vascular smooth muscle. J. Physiol..

[42] Brozovich F.V., Nicholson C.J., Degen C.V., Gao Y.Z., Aggarwal M., Morgan K.G. (2016). Mechanisms of vascular smooth muscle contraction and the basis for pharmacologic treatment of smooth muscle disorders. Pharmacol. Rev..

[43] Saphirstein R.J., Gao Y.Z., Jensen M.H., Gallant C.M., Vetterkind S., Moore J.R., Morgan K.G. (2013). The focal adhesion: A regulated component of aortic stiffness. PLoS ONE.

[44] Turk M., Baumeister W. (2020). The promise and the challenges of cryo-electron tomography. FEBS Lett..

[45] Burbaum L., Schneider J., Scholze S., Böttcher R.T., Baumeister W., Schwille P., Plitzko J.M., Jasnin M. (2021). Molecular-scale visualization of sarcomere contraction within native cardiomyocytes. Nat. Commun..

[46] Tacke S., Erdmann P., Wang Z., Klumpe S., Grange M., Plitzko J., Raunser S. (2021). A streamlined workflow for automated cryo focused ion beam milling. J. Struct. Biol..

[47] Wang Z., Grange M., Wagner T., Kho A.L., Gautel M., Raunser S. (2021). The molecular basis for sarcomere organization in vertebrate skeletal muscle. Cell.

[48] Fox J.G., Schultz C.S., Boler B.M.V., Fox J.G., Marini R.P. (2014). Nutrition of the Ferret. Biology and Diseases of the Ferret.

[49] Flanagan L.A., Chou J., Falet H., Neujahr R., Hartwig J.H., Stossel T.P. (2001). Filamin A, the Arp2/3 complex, and the morphology and function of cortical actin filaments in human melanoma cells. J. Cell Biol..

